# A Rare Case Presentation on Total Colonic Aganglionosis in a Female Infant of Indian Origin

**DOI:** 10.7759/cureus.49847

**Published:** 2023-12-02

**Authors:** Krushank Nayak, Kiran Khedkar, Rajesh G Gattani, Raju K Shinde, Shubham Durge, Dhaval Patel

**Affiliations:** 1 General Surgery, Jawaharlal Nehru Medical College, Datta Meghe Institute of Higher Education & Research, Wardha, IND; 2 Pediatric Surgery, Jawaharlal Nehru Medical College, Datta Meghe Institute of Higher Education & Research, Wardha, IND

**Keywords:** colonic hypoganglionosis, hirschsprung's disease, transverse colon hypoganglionosis, distal ileum hypoganglionosis, total colonic aganglionosis

## Abstract

Total colonic aganglionosis, also called total colonic Hirschsprung's disease, is a known congenital disorder caused by the migration of abnormal embryonic neuroblasts. RET, NRG1, and L1CAM genes are reported as pathological gene variants associated with the incidence of different variants of Hirschsprung's disease. Major clinical presentations are well documented as inefficiency to pass stools, vomiting, fever, persistent crying, and other features of intestinal obstruction. We present here the case of a two-day-old female infant of Indian origin and its diagnostic, clinical, and case management data.

## Introduction

Total colonic aganglionosis is characterized by the absence of a nerve plexus in the muscular layer of the large intestine, preventing it from relaxing properly. This leads to continuous constriction of the large intestine, impeding the normal passage of gas and stools. In some cases, especially in children, it is not immediately apparent, as they may pass some stools despite the condition, and the diagnosis may be delayed until later in life when distal involvement becomes evident. Histologically and clinically, total colonic aganglionosis has been identified as a variant of Hirschsprung's disease [1]. Various therapeutic management procedures for addressing this condition are documented in the research literature [2]. It has predominantly been observed in males [3,4]. Here, we present a case involving a female infant diagnosed with total colonic aganglionosis.

Hirschsprung's disease exhibits a higher prevalence in males, with a male-to-female ratio of approximately 4:1 [2,5]. However, in cases of long-segment disease, this ratio decreases to 2:1 [2]. In instances of long-segment Hirschsprung's disease, the gender ratio evens out to 1:1 [3]. In a sample of Hirschsprung's disease patients, there were 14 females for every 25 males [1]. Boys diagnosed with Hirschsprung's disease experienced more frequent hospitalizations and a higher incidence of abnormal defecation frequency compared to girls with the condition [4].

## Case presentation

**Figure 1:**
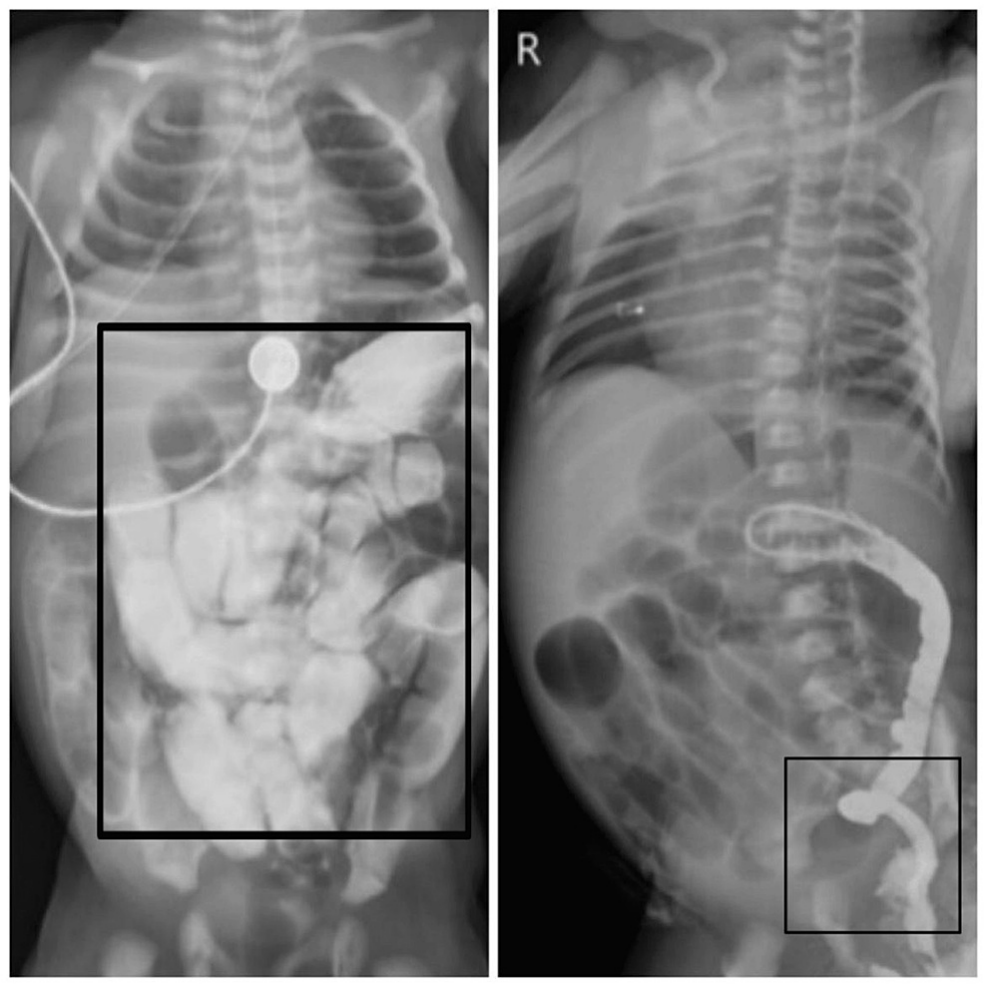
Contrast dye study suggests narrowing of the descending colon and multiple air fluid levels. L and R demonstrating collapse of the large intestine L: anterior; R: lateral

A 3-kg female infant delivered through a normal vaginal delivery from a G1P1L1 mother was referred for specialized care. The primary concerns upon presentatiostive of hypogangln included bilious vomiting after the initial feed and a lack of bowel movements since birth. An abdominal X-ray was conducted, revealing indications of dilated bowel loops. Upon admission, in-house ultrasonography was performed, indicating the presence of multiple air fluid levels and a dilated bowel loop with fecal matter in the anal canal. Following consultation with the general surgery team, a dye study revealed dilation in the small bowel, as illustrated in Figure 1.

**Figure 2:**
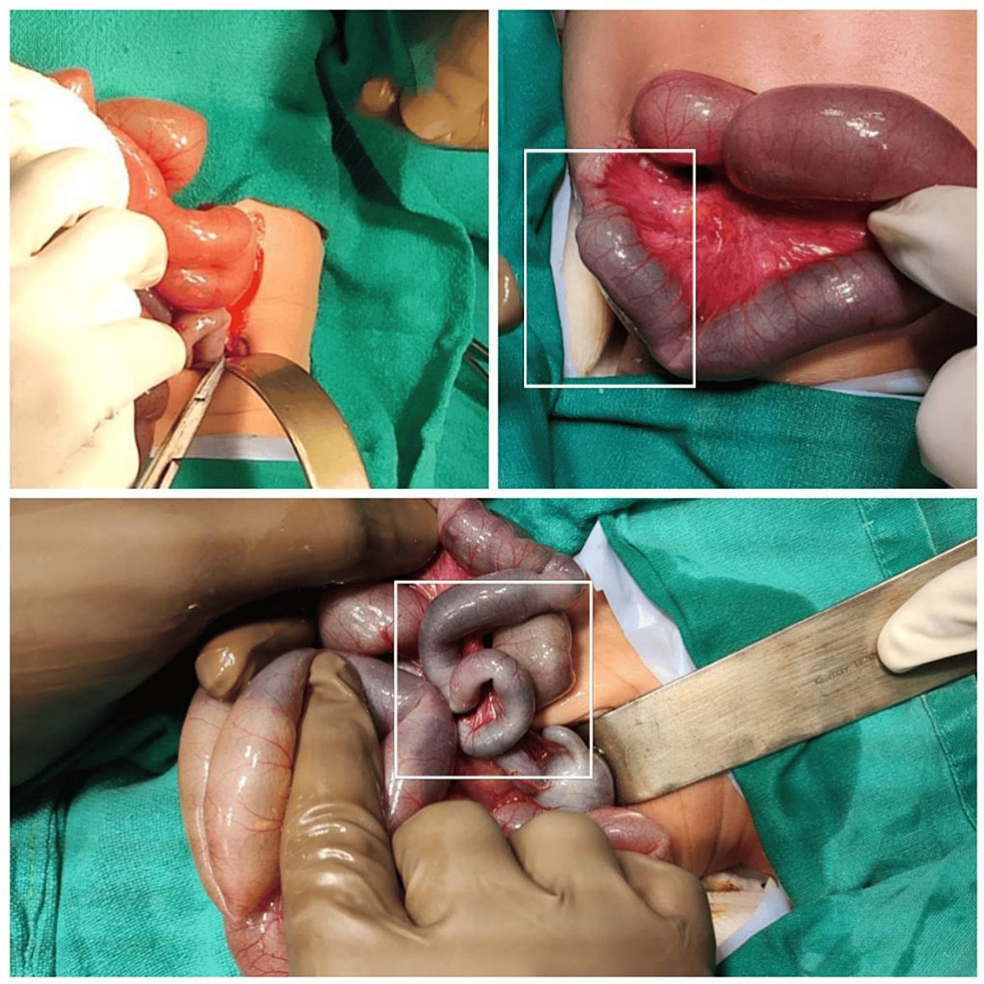
Intraoperative photographs suggestive of narrowing of the colon with transition zones

Following the dye study, an emergency exploratory laparotomy was conducted. During the intraoperative examination, constriction of the large intestine was observed extending up to the ileocecal junction. Additionally, a transition zone was identified on the ileum, as depicted in Figure 2.

**Figure 3:**
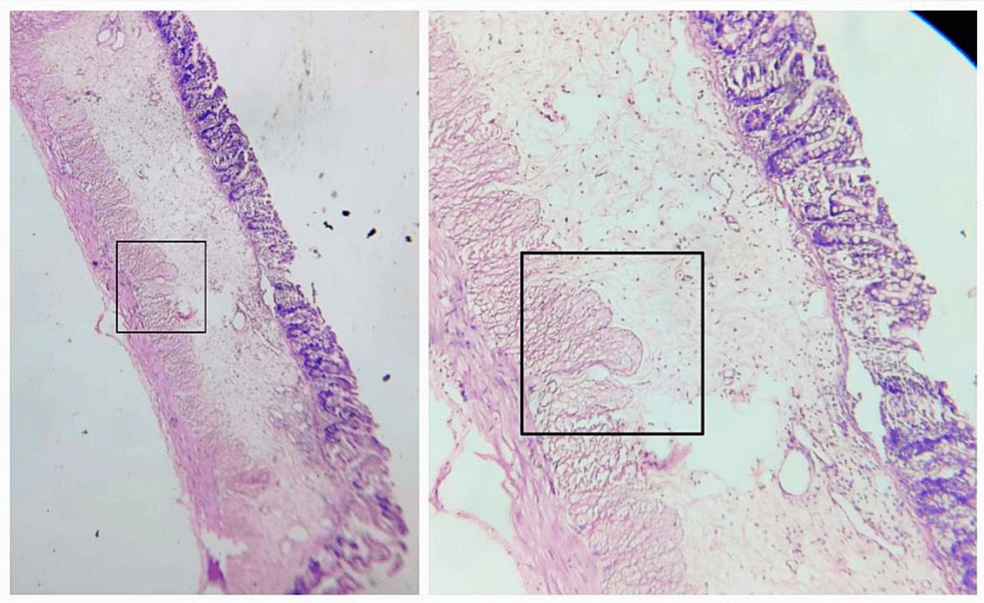
Histopathology images of hematoxylin and eosin-stained section suggestive of hypoganglionic cell till ileocecal junction

As a result, an ileostomy was performed, and biopsy samples were collected from various sites in the colon. These samples were then sent for additional histopathological examination. The histopathology report indicated the absence of a nerve plexus up to the ileocecal junction, as illustrated in Figure 3.

The primary post-ileostomy complication was diarrhea, stemming from insufficient water absorption in the gut mucosa. Following the patient's stabilization, the recommended course of action was definitive surgery, considered the primary curative treatment. The surgical intervention proposed encompassed a total colectomy involving the removal of the terminal ileum and the stoma part of the ileostomy. A pull-through procedure was also suggested, wherein a segment of healthy ganglionic cell-containing ileum would be connected to the rectum using the Duhamel procedure. Consequently, an emergency laparotomy was planned. Intraoperative observations revealed a narrowing extending throughout the entire large colon, with a transition zone identified at the distal ileum, approximately 3 cm from the ileocecal junction. Subsequently, an ileostomy was scheduled, accompanied by the collection of multiple biopsy samples from various segments of the large intestine, including the transition zone and distal ileum. The histopathology report confirmed the absence of a nerve plexus up to the transition zone. As part of the planned approach, the definitive procedure was deferred until one year after the ileostomy, allowing for adequate patient stabilization and preparation for the subsequent surgical intervention.

## Discussion

Hirschsprung's disease exhibits a higher prevalence in males, with a male-to-female ratio of approximately 4:1 [5]. The diagnosis of total colonic aganglionosis poses a challenge due to its rarity, leading to varying approaches among surgeons and pathologists. A definitive diagnosis requires comparing histopathological and radiological findings with clinical presentations [1]. Hirschsprung's disease, which can also be congenital, has been associated with disruptions in the normal colonic and fecal microbiome [6]. Cases of total colonic aganglionosis in female infants are exceedingly rare [7], and their diagnosis may be delayed due to the invisibility of transition zones.

A recent case report highlighted a rare instance of total colonic aganglionosis in a Caucasian infant, diagnosed retroactively [8]. Diagnosis based on histological findings from intestinal sections, ileum, and appendix biopsy samples remains a topic of debate among researchers [8,9]. Some researchers advocate for caution in the diagnosis, expressing reservations about Hirschsprung's disease, emphasizing the importance of consistent clinical presentations irrespective of radiological and biopsy findings [3].

As a congenital disorder, total colonic aganglionosis has reported genetic variations. Major risk alleles linked to the incidence of Hirschsprung's disease include RET, EDNRB, ZFHX1B, Trisomy 21, and Sox 10. A whole genome sequence analysis study in an Asian population proposed an association of BACE2 (OR: 7.3) with Hirschsprung's disease based on sequencing result inferences from an ethnically matched case-control study [10]. Aligning with recent research by Chakhunashvili et al. and other scholars [3,11], we advocate for a comprehensive diagnostic approach and a thorough risk factor analysis in the case management and diagnosis of total colonic aganglionosis.

## Conclusions

Our case of total colonic aganglionosis in a female infant contributes valuable insights to the current body of literature on Hirschsprung's disease. While traditionally considered more prevalent in males, our case underscores the importance of recognizing and scrutinizing rare instances in female infants. The early presentation of this condition after birth necessitates a heightened awareness in clinical settings. The outcomes in total colonic aganglionosis, as observed in our case, emphasize the significance of a timely and accurate diagnosis. The integration of clinical, radiological, and laboratory findings, coupled with a comprehensive management approach, has proven effective in achieving satisfactory outcomes. Our case underscores the need for a nuanced understanding of the disease spectrum, especially in females, and emphasizes the critical role of a multidisciplinary approach in optimizing patient care. This contribution aims to further inform healthcare professionals, researchers, and clinicians, providing additional perspectives on the diagnosis and management of total colonic aganglionosis, particularly in female infants. Our findings support the evolving understanding of the disease and underscore the importance of ongoing research to enhance diagnostic accuracy and optimize patient outcomes.
